# Awareness about antibiotic resistance in a self-medication user group from Eastern Romania: a pilot study

**DOI:** 10.7717/peerj.3803

**Published:** 2017-09-12

**Authors:** Gabi Topor, Ionela-Alina Grosu, Cristina Mihaela Ghiciuc, Aurel Lulu Strat, Cătălina Elena Lupuşoru

**Affiliations:** 1 Department of Pharmacology—Morphofunctional Sciences II, University of Medicine and Pharmacy Grigore T. Popa, Iasi, Romania; 2 Laboratory of Microbiology, Hospital of Infectious Diseases Saint Parascheva, Iasi, Romania

**Keywords:** Antibiotic bacterial resistance, Self medication users, Practices, Awareness, Attitudes, Health knowledge, General population, Medical residents

## Abstract

**Background:**

Awareness about antibiotic resistance depends on the attitudes and information about antibiotic resistance of both patients and physicians. Persons who practice self-medication are at high risk of also self-medicating with antibiotics. The purpose of the present study was to evaluate the awareness about antibiotic resistance by investigating the practice in a group of self-medication users in a sample of adults in Romania and the variables associated with such practice.

**Material and Methods:**

A cross-sectional self-filled questionnaire based study was conducted from December 2016 through January 2017 amongst 218 self-medication users (SMUG). The attitudes, the level of knowledge, the perceptions, about antibiotic use (ABU) and about antibiotic resistance (ABR) were compared to a reference group represented by medical residents group in their specialty training (MRG) considered to have a higher level of knowledge and awareness about ABU and ABR.

**Results:**

The response rate was 87.2% in the SMUG group and 100% in the MRG group. The SMUG group reported self-medication practices for antibiotics with a high frequency at any time in life (72%), but with a very low frequency from the month previous to the date of the study (12%), comparative with the MRG group (75% and 7%, respectively). The multivariate logistic regression analysis showed that self-medication with antibiotics at any time in life in the SMUG group could be predicted by the answers to two questions regarding the practices and knowledge about ABU (Q13 and Q20). On the other hand, in the MRG group, a question about ABR perception (Q23), could be predictor for self-medication with antibiotics. Self-medication with antibiotics in the month previous to the date of the study in the SMUG group could be predicted with three questions: one about ABU practice (Q14), one about ABR perception (Q26) and one referring to ABR knowledge (Q28). On the other hand, in the MRG group, a question about ABR knowledge (Q32) could be predictor for self-medication with antibiotics. The reduced awareness about ABR in the SMUG group, is revealed by the reduced number of subjects (38%), who did not know that missing an antibiotic dose during a medical treatment contributes to ABR, comparative with the MRG group (84%). Indirectly, low ABR awareness in the SMUG group is revealed by the confusion about the appropriate use of antibiotics in bacterial or viral infections (that antibiotics are not used against viruses).

**Conclusions:**

The findings from our study on the awareness about antibiotic resistance in the SMUG group might help the policy makers and regulatory authorities to develop educational programs directed to change the perceptions and attitudes about the appropriate use of antibiotics in order to diminish self-medication practices with antibiotics.

## Introduction

Inappropriate use of antimicrobial drugs is closely related to the knowledge, attitudes and behavior of the population, as well as the antibiotic prescribing behavior of the healthcare professionals, all these contributing to the increase of antibiotic resistance (ABR) (Gualano et al., 2015). Improvement of the rational use of antimicrobials is encouraged by the World Health Organization through prescription-only use of antibiotics (Leung et al., 2011) and through educational measures (Lee et al., 2015).

The frequency of antibiotic use places Romania on the first place for antibiotic self-medication and on the second place regarding total antibiotic consumption in the European Community, according to a recent report from the European Centre for Disease Prevention and Control of the latest data of antibiotic consumption in the European Union (Centre for Disease Prevention and Control, 2016). In 2009, “Romania was the only European country where fewer than eight out of ten citizens had obtained antibiotics from a doctor (79%)”, according to European Commission, 2009. As a result of a large number and variety of interventions at national level initiated to promote the prudent use of antibiotics in Romania, a recent survey from June 2016, requested by the European Commission Directorate-General for Health and Food Safety and coordinated by the Directorate-General for Communication, found that 84% of the Romanian population obtained antibiotics from a doctor (European Commission, 2016). Therefore, there is a need for new studies to evaluate particular aspects of awareness about ABR.

Self-medication practices with “over the counter” or other groups of drugs (analgesics/antipyretics, gastro-intestinal drugs, respiratory drugs, vitamins) is of benefit in the timely management of common illnesses; the prevalence varies in different countries or minorities groups as was shown by several surveys conducted in different geographic areas (Alexa et al., 2014; Berzanskyte et al., 2006; Eticha & Mesfin, 2014; European Commission, 2013; Garofalo, Di Giuseppe & Angelillo, 2015; Jafari, Khatony & Rahmani, 2015; Martin-Perez, Barrera & Andreas, 2015; Ocan et al., 2014), but self-medication practices with antibiotics (administration of antibiotics without a medical prescription) increases the risk of ABR. Therefore, analyzing this practice is needed in order to identify factors that can reduce the consequences of inappropriate antibiotics use. Only a few studies were conducted to assess the magnitude of self-medication practices with antibiotics among self-medication users (Grigoryan et al., 2008; Landers et al., 2010; Muras et al., 2013; Selvaraj, Kumar & Ramalingam, 2014) and only a reduced amount of information is available about this phenomenon in Romania (Anghel & Craciun, 2013; Bungău et al., 2015; Voidazan et al., 2016).

Attitudes, perceptions, knowledge and beliefs about antibiotic use (ABU) and ABR were investigated by a number of cross-sectional studies (André et al., 2010; Grigoryan et al., 2007; Ivanovska et al., 2013), conducted either on groups from the general population or on groups of individuals with different levels of medical knowledge. The level of education, age, or other factors influence the knowledge and the attitudes of the general population and vary according to the countries where they had been conducted: Sweden (Grigoryan et al., 2007; Vallin et al., 2016), Italy (Napolitano et al., 2013), Spain (Mira et al., 2014) or Greece (Skliros et al., 2010). The are also studies assessing the clinician’s or general population’s knowledge and beliefs concerning the awareness about ABR (Bert et al., 2017; McCullough et al., 2015; Muras et al., 2013; Paget et al., 2017; El Zowalaty et al., 2016), but there are less studies concerning the awareness about ABR in particular population groups. The impact of predisposing factors (e.g., attitudes and knowledge concerning antibiotic use and self-medication) on self-medication with antibiotics in many European countries was studied by many projects such as Self-Medication with Antibiotics and Resistance Levels in Europe (SAR) or European Surveillance of Antimicrobial Consumption (ESAC) (Adriaenssens et al., 2015; Muscat et al., 2006; Olczak et al., 2006; Versporten et al., 2014).

Awareness about ABR depends on the attitudes and information about ABR of both patients and physicians. The evaluated parameters for awareness about ABR were: the attitudes, the level of knowledge and the perceptions about ABU and/or ABR. The purposes of the present study was to evaluate the awareness about antibiotic resistance by investigating the practice in a group of self-medication users in a sample of adult in Romania and the variables associated with such practice.

## Materials and Methods

### Ethics statement

The study was approved by University of Medicine and Pharmacy Gr. T. Popa Iasi Ethics Committee. Written informed consent was considered not to be necessary, since the study evaluated only the knowledge, attitudes, perceptions and practices of the participants.

### Self-medication user group (SMUG), study site and sampling

This cross-sectional self-filled questionnaire based study was conducted in the winter months, from December 2016 through January 2017. Self-medication user group (SMUG) from our study are defined as individuals who addressed to the pharmacy in order to buy any medication without having a prescription. The study was designed to sample randomly at least 215 subjects over 18 years old from the SMUG group. Four private pharmacies from Galati, one of the largest cities in the East of Romania, similar in terms of size and patient load per month, located in different areas of the city were selected (from a list of all centers, we called randomly and selected the first four which accepted to enter the study). We asked every other third person that entered the pharmacy if it came with the purpose of buying medication without having a prescription; if the answer was positive we randomly choose her by coin tossing (heads = request her; tails = not request her) to participate in the study. Subjects with medical training were excluded. The subjects who accepted to participate were informed about the purpose of the study and assured of the confidentiality of the results. After obtaining their verbal consent for the participation in the study, they received the self-administered questionnaire. The questionnaires did not contain personally identifiable information, but only a code number as identifier. Data were collected by a team of PhD students, trained to answer to the participant’s queries about the questionnaire, if any. A reference group, represented by 100 medical residents in their specialty training (MRG), was randomly selected to answer to the same questionnaire being considered to have a higher level of knowledge and awareness about ABU and ABR by comparison to the general population.

### Sample size

We considered that the prevalence of non-prescription antibiotic use in Romania is 17%, based on the data from 2016 (European Commission, 2016). Raosoft^®^ software was used to compute the minimum required sample size. If the non-prescription antibiotic use prevalence is 17%, to obtain a margin of error of 0.05%, with a 95% confidence interval, the sample size needed would be minimum 215 participants. To adjust for possible missing in filling of the data from questionnaire, we selected 250 participants for the SMUG group. For the MRG group, based on the calculation that the prevalence of non-responders in a class survey is about less than 5%, to obtain a margin of error of 0.05%, with a 95% confidence interval, the minimum sample size needed would be 73 participants, so we selected 100.

### Development of the questionnaire

A self-administered questionnaire written in Romanian was compiled after a detailed review of relevant literature (McCullough et al., 2015; Shehadeh et al., 2012), taking into account that both the general population and medical residents in training were supposed to answer to the same questions. The questionnaire had 37 questions with closed or open answers that evaluated the practices, knowledge, perceptions and attitudes of the population under study. Scores were created by summing the scores for respective items such that a higher score indicated more positive perceptions and attitudes about ABR. For the Perceptions about ABR, M = 6.89, SD = 2.55, and Cronbach’s Alpha (an estimate of internal consistency and scale reliability) was 0.70. For Attitudes about the ABR, M = 7.01, SD = 2.51, and Cronbach’s Alpha was 0.69. The questionnaire was tested on a group of 20 final year medical students and on 20 subjects selected from the patients coming to a pharmacy to identify any ambiguity in the questions and validated it on two other similar groups. Data collected during this validation pilot study were excluded from the final analysis.

The questionnaire had six sections:

• ***Participant’s characteristics*** were collected by seven questions: age, gender, place of residence (rural/urban), level of education (undergraduate/graduate), occupation (employed/unemployed), together with the self-evaluation of responder’s health status and the attitude in the case of a health problem.
• ***Practice about ABU*** was evaluated by seven yes/no type questions. The responders were asked to indicate prior ABU at any time in life and separately, in the month previous to the date of the study. They were also asked to specify if they intend to follow the indications of the physician regarding the recommended antibiotic treatment, about their intention to demand an antibiotic treatment in the case of a common cold or dental infection, if they are interested in obtaining information about the recommended antibiotic.
• ***Basic knowledge about antibiotic use*** was evaluated by two yes/no/“I don’t know” type questions, four true/false type questions and two multiple choice type questions with only one correct answer. These questions evaluated the knowledge about antibiotic safe use and about the use of antibiotics only in bacterial and not in viral infections.
• ***Perception about the phenomenon of ABR*** was evaluated by five yes/no/“I don’t know” type questions about ABR as a problem at national and hospital level or for the future of the medical practice.
• ***Basic knowledge about ABR*** was evaluated by one yes/no/“I don’t know” type question about ABR as a consequence of missing one dose of antibiotic, and four true/false type questions about the definition of ABR and about the factors contributing to ABR.
• ***Attitudes about the measures used by the physician to prevent ABR*** were evaluated by five questions based on the 5-point Likert scale (strongly agree/agree/neutral/disagree/strongly disagree) about the agreement with some of the measures used by the physician to prevent ABR.

### Data analysis

Data were coded and analyzed using SPSS^®^ v.11 software for Windows (SPSS Inc., Chicago, IL, USA). The Likert’s items were combined and reduced into two categories: “strongly agree, agree” =Agree and “neutral, disagree, strongly disagree” = Disagree; for the other type of answers “yes” = Yes and “no, I don’t know”= No. Descriptive statistics were used to summarize the numerical variables (mean and standard deviation) and categorical variables (expressed as frequencies and percentages). Categorical variables were assessed by chi-square test or Fisher’s exact test when appropriate. Continuous variables were assessed for statistical significance using the Kruskal–Wallis One Way Analysis of Variance on Ranks. A multivariate logistic regression analysis with antibiotic self-medication as the dependent variable was performed to evaluate the relationship with the attitudes, perceptions and the knowledge level of the responders. Those variables achieving in the bivariate analysis a *p* value ≤0.1 were included in the multivariate logistic regression models, using stepwise selection of variables, to examine for the effects of each independent variable on the different outcomes of interest. The criterion for entering and exiting the variables in the model was, respectively, being of *p* value ≤0.1 and *p* value ≤0.05. Two multivariate logistic regression models have been constructed: self-medication with antibiotics at any time in life (Model 1) and self-medication with antibiotics in the last month (Model 2). The variables represented by the answers to the following questions: Q1–3 and Q10–37 were included in all models. The results of the multivariate logistic regression models are presented as adjusted odds ratios (ORs) along with their 95% confidence intervals (CIs) and the percent concordant. A probability level of *p* ≤ 0.05 was considered as denoting statistical significance.

## Results

### Participants’ characteristics

By the end of the data collection phase, 218 fully completed questionnaires out of 250 of the SMUG group were collected, and 32 incomplete questionnaires were removed and not included in the data analysis. All 100 questionnaires from the MRG group were fully filled. The response rate was 87.2% in the SMUG group and 100% in the MRG group. The mean age in the SMUG group was 40 ± 12 years and in the MRG group was 29 ± 3 years. In the SMUG group, 82% were employed and 86% were urban residents. The SMUG group was composed of 69% females, while in the MRG group 75% were females. In the SMUG group, 72% called the family doctor, 14% called a specialized doctor as the first person in the case of a health problem, while 8% called the pharmacist and 6% self-medicated. In the MRG group, 51% called the family doctor, 26% called a specialized doctor as the first person in the case of a health problem, while 23% self-medicated. No further analysis of non-respondents was conducted.

### Antibiotic use (ABU): practices and basic knowledge

Practices and basic knowledge about ABU are shown in the Table 1.

**Table 1:** ABU: practices and basic knowledge.

	Answer considered correct	No (%)		*p*-value
		SMUG (*n* = 218)	MRG (*n* = 100)	
**Practices about ABU**				
Q8: Self-medication with antibiotics at any time in life.	Yes	156 (72%)	75 (75%)	NS
Q9: Self-medication with antibiotics in the last month.	Yes	27 (12%)	7 (7%)	NS
Q10: Compliant with the physician recommendation regarding the antibiotic treatment.	Yes	207 (95%)	99 (99%)	NS
Q11: Return to the physician after the antibiotic treatment.	Yes	99 (45%)	44 (44%)	NS
Q12: Demand an antibiotic treatment for a common cold.	No	175 (80%)	97 (97%)	<0.001
Q13: Demand an antibiotic treatment for a dental infection.	Yes	182 (83%)	99 (99%)	<0.001
Q14: Interested to ask for information about the prescribed antibiotic.	Yes	175 (80%)	94 (94%)	0.003
**Basic knowledge about ABU**				
Q15: Antibiotic administration may induce adverse effects.	Yes	192 (88%)	100 (100%)	0.002
Q16: Some antibiotics may induce malformations.	Yes	106 (49%)	97 (97%)	<0.001
Q17: Bacteria may cause common cold.	False	120 (55%)	96 (96%)	<0.001
Q18: Antibiotics are efficient for the treatment of bacterial infections.	True	166 (76%)	100 (100%)	<0.001
Q19: Antibiotics are efficient for the treatment of viral infections.	False	161 (74%)	100 (100%)	<0.001
Q20: Antibiotics are efficient for the treatment of both bacterial and viral infections.	False	131 (60%)	98 (98%)	<0.001
Q21: The usual duration of antibiotic treatment in pneumonia (<3 days, 3–7 days, >7 days).	3–7 days	173 (80%)	92 (92%)	0.008
Q22: Choose the situation which needs antibiotic treatment (cough, chills, inflammation, dental infection, throat, pain, fever, neoplasia).	Dental infection	152 (70%)	87 (87%)	0.002

**Notes.**

SMUG self-medication user group MRG medical residents group

Data are expressed as number and response rate for the correct answer. Statistical analysis: chi-square test.

Concerning the practices about ABU, the responders from the two study groups reported similar self-medication practices for ABU. The subjects reported self medication practices with a high frequency at any time in life, but with very low frequency from the month previous to the date of the study. Although almost all responders agreed to follow the physician’s recommendations regarding antibiotic treatment (95% of the SMUG group and 99% of the MRG group), only 45% of the SMUG group and 44% of the MRG group were willing to return to check with the physician after the antibiotic treatment. Many individuals from the SMUG group had inappropriate practices about ABU, based on the intention to ask for an antibiotic treatment in the case of a common cold (only 80% answered correctly “No”) or in the case of a dental infection (only 83% answered correctly “Yes”), respectively to ask for information about the prescribed antibiotic (80%). More than 94% of the MRG group had very good ABU practices evaluated by the answers to these questions.

Basic knowledge about the use of antibiotics was of medium level in the SMUG group. Confusion regarding some questions about the appropriate use of antibiotics (Q15, Q16, Q17, Q18, and Q22) is high among the SMUG group. Inaccurate knowledge about the effectiveness of antibiotics on bacteria or on viruses was higher among the SMUG group (only 60% responded correctly), although most of the respondents (80% in the SMUG group) knew the usual duration of antibiotic treatment for pneumonia. The usual duration of the treatment for pneumonia and the necessity of an antibiotic treatment were not clearly known but by a few of the SMUG group and this appropriate knowledge existed to nearly all of the MRG.

### ABR: perceptions, basic knowledge and attitudes

Awareness about ABR is lower among the subjects from the SMUG group, as it is presented in the Table 2.

**Table 2:** ABR: perceptions, basic knowledge and attitudes.

	Answer considered correct	No (%)		*p*-value
		SMUG (*n* = 218)	MRG (*n* = 100)	
**Perceptions about ABR**				
Q23: Do you believe that the antibiotics you will receive might contribute to ABR?	Yes	94 (43%)	43 (43%)	0.007
Q24: As a future problem for the medical practice.	Yes	147 (67%)	84 (84%)	<0.001
Q25: Do you think that it is better to take less antibiotics than those prescribed?	No	110 (50%)	48 (48%)	0.017
Q26: As a national problem.	Yes	130 (60%)	95 (95%)	<0.001
Q27: As a problem for Romanian hospitals.	Yes	142 (65%)	99 (99%)	<0.001
**Basic knowledge about ABR**				
Q28: Missing an antibiotic dose contributes to ABR.	Yes	82 (38%)	84 (84%)	<0.001
Q29: ABR is a result of insufficient knowledge about ABU.	True	197 (90%)	100 (100%)	0.003
Q30: ABR defined as if taken too often, antibiotics will become less and less effective in the future.	True	158 (72%)	98 (98%)	<0.001
Q31: ABR is caused by the overuse of antibiotics.	True	189 (87%)	92 (92%)	NS
Q32: ABR can result after inappropriate use of antibiotics outside the doctor’s indications.	True	203 (93%)	95 (95%)	NS
**Attitudes about the ABR**		Strongly / Partially Agreed (%)		
Q33: Waiting for the laboratory results in severe infections		194 (90%)	100 (100%)	<0.001
Q34: Administering an antibiotic in a febrile patient unless severity criteria are present		175 (80%)	93 (93%)	<0.001
Q35: Accepting the most frequent recommended antibiotic		210 (97%)	100 (100%)	0.017
Q36: Respecting the dose and timing of administration		209 (96%)	100 (100%)	<0.001
Q37: Respecting the antibiotic duration of treatment		190 (87%)	100 (100%)	0.002

**Notes.**

SMUG self-medication user group MRG medical residents group

Data are expressed as number and response rate for the correct answer. Statistical analysis: chi-square test.

A big confusion in the perception of ABR was noticed among the responders from both study groups. Two questions, one regarding the contribution of any antibiotic to ABR (Q23) and another one regarding the preference for taking less antibiotics than those prescribed (Q25), have quite similar frequencies of responders in both groups and the chi-square test showed statistically significant differences between the study groups. ABR was not perceived as a future problem for medical practice by 67% of the SMUG group and by 84% of the MRG group. On the contrary, almost all in the MRG group were aware that ABR is a national problem, but they perceived ABR more as a hospital linked problem rather than a national problem.

Basic knowledge about ABR was weaker in the SMUG group compared to the MRG group. The statement that a missed dose of antibiotic during the treatment could contribute to ABR it was known only by 38% of the SMUG group and by 84% of the MRG group. From the SMUG group 72% defined correctly ABR, although 87% and 93% knew that ABR could result because of the overuse or inappropriate use of antibiotics outside the doctor’s indications, respectively.

Inappropriate attitudes about the measures to prevent ABR were noticed in a small number of responders from the SMUG group. The great majority of the responders from SMUG group agreed with the measures to prevent ABR. The decision for prescribing or not of antibiotic in a febrile patient unless severity criteria are fulfilled was not agreed by 7% of the MRG group.

### Basic knowledge scores

The scores were computed as follows: 0–8 for knowledge about ABU and 0–5 for knowledge about ABR. The accurate knowledge about antibiotics was evaluated based on the maximum cumulated scores of the questions regarding ABU and ABR and on the correct answers to the two key questions: one about the duration of antibiotic treatment for bacterial pneumonia (Q21) and one about the indications for antibiotic prescription in other pathological situations (Q22).

According to our cumulative scoring, the mean accurate score for the SMUG group was 2.09 ± 1.1, statistically significantly lower compared to the MRG group (3.58 ± 0.6; *p* < 0.05).

Using Q22 as control question for Q13, we notice that the matching for the correct answer to both questions (a subject should answer to both questions correctly, if having correct knowledge and correct practice) was only 58% in the SMUG group by comparison to 81% in the MRG group. This could mean that it is possible that 26% in the SMUG group by comparison to 8% in the MRG group have mistaken inflammation for infection.

### Multivariate logistic regression analysis of potentially predictive factors associated with self-medication practices

The multivariate logistic regression analysis showed that self-medication with antibiotics at any time in life or in the month before the study could be potentially predicted by the independent variables represented by the coded answers to the following questions: (Table 3).

**Table 3:** Regression models for potential determinants of the different outcomes of interest.

Variable	OR	95% CIs	Percent concor-dant (%)	*p*-value
**Model 1. Self-medication with antibiotics at any time in life (Q8).**				
SMUG (*n* = 218)				
Q13: Demand an antibiotic treatment for a dental infection.	0.38	0.18–0.80	46	0.011
Q20: Antibiotics are efficient for the treatment of both bacterial and viral infections.	0.48	0.25–0.91		0.026
MRG (*n* = 100)				
Q23: Do you believe that the antibiotics you will receive might contribute to ABR?	0.41	0.23–0.75	54	0.004
**Model 2. Self-medication with antibiotics in the last month (Q9).**				
SMUG (*n* = 218)				
Q14: Interested to ask information about the prescribed antibiotic.	0.24	0.09–0.58	71	0.002
Q26: ABR is a national problem.	2.02	1.18–3.45		0.010
Q28: Missing an antibiotic dose contributes to ABR.	0.45	0.26–0.80		0.007
MRG (*n* = 100)				
Q32: ABR can result after inappropriate use of antibiotics outside the doctor’s indications.	34.12	4.39–265.16	42	<0.001

**Notes.**

Statistical analysis: multivariate logistic regression analysis.

In the Analysis of Maximum Likelihood Estimates, the positive or negative influence the independent variables selected in the models have on the dependent variables (self-medication) are indicated by the sign of the estimated coefficients. The modeled probability is self-medication = 1, the event. For the coding of the variables, see the Supplemental Information.

Model 1: In the SMUG group the answers to two questions regarding the practices and knowledge about ABU (Q13: “*Demand an antibiotic treatment for a dental infection.*” and Q20: “*Antibiotics are efficient for the treatment of both bacterial and viral infections.*”) could be negative potential predictors (negative sign for estimated coefficient) for self-medication at any time in life, with the correct answer: Q13 = yes and Q20 = no. Because of less knowledge in the SMUG group (possibly inflammation mistaken for infection at Q13 and no effect of antibiotics in viral infections and/or viral infections mistaken for bacterial infections at Q20) by comparison to the MRG group, the frequency of correct answers is less (Table 1).

Model 2: In the MRG group, a question referring to the perception about ABR (Q23: “*Do you believe that the antibiotics you will receive might contribute to ABR?*”) could be a negative potential predictor for self-medication at any time in life with antibiotics, with the correct answer: Q23 = yes.

Self-medication with antibiotics in the month previous to the date of the study in SMUG group could potentially be predicted with three questions: one about ABU practice (Q14: “*Interested to ask information about the prescribed antibiotic.*”) as a negative potential predictor, with the correct answer: Q14 = yes; one about ABR perception (Q26: “*ABR is a national problem.*”) as a positive potential predictor, with the correct answer: Q26 = yes, and one referring to ABR knowledge (Q28 “*Missing an antibiotic dose during the treatment contributes to ABR.*”) as a negative potential predictor, with the correct answer: Q28 = yes. The positive potential predictor at Q26, which came up in the model for the SMUG group, was thought to be due to the insufficient informational campaign at the national level concerning self-medication and antibiotic resistance, which was reflected in the low frequency of correct answers in the SMUG group by comparison to the MRG group (Table 2).

In the MRG group, a question referring to the knowledge about ABR (Q32: “*ABR can result after inappropriate use of antibiotics outside the doctor’s indications.*”) could be a potential positive predictor for self-medication with antibiotics in the month previous to the date of the study, with the correct answer: Q32 = yes.

## Discussion

Our study found inadequate awareness about ABR along with inappropriate practices, attitudes and knowledge about ABU and ABR in the SMUG group and also in the reference group of MRG.

In the SMUG group, self-medication with antibiotics at any time in life was 72%, more than in another study which reported self-medication with antibiotics in 67.2% of adults from the Northern Manhattan (Landers et al., 2010). Interestingly, in the reference group of MRG, self-medication with antibiotics at any time in life was almost similar: 75% (differences were not statistically significant). We cannot consider appropriate self-prescription in the MRG group (prudent and appropriate use) instead of self-medication, because they declared themselves that they took antibiotics without prescription (self-medication). Our study did not evaluated self-prescribing in the MRG group as it was described in a Norwegian study (Hem et al., 2005) that found antibiotics as the most frequently self-prescribed medications (71%–81%). Self-prescribing is acceptable in some situations for physicians, but in the case of antibiotics they should seek professional help for illness. There are only a few data available about self-medication with antibiotics from Romanian studies. (Anghel & Craciun, 2013; Bungău et al., 2015; Damian, Lupuşoru & Ghiciuc, 2014; Voidazan et al., 2016). Our study confirms the findings of higher prevalence of self-medication among educated subjects (Gualano et al., 2015; Grigoryan et al., 2007; André et al., 2010; Vallin et al., 2016).

Self-medication with antibiotics reported from the month previous to the date of the study, showed lower and quite similar percentages (12% in the SMUG group, respectively 7% in the MRG group); the differences were not statistically significant. Reported from other time intervals (last 12 months, last six months), self-medication use was found higher in Greece (45%), Romania (44%) and Croatia (32%) compared to other countries in Western Europe (Skliros et al., 2010; Grigoryan et al., 2006; Damian, Lupuşoru & Ghiciuc, 2014; WHO, 2014; Paget et al., 2017). The “Antimicrobial resistance and the causes of non-prudent use of antibiotics” (ARNA) project that specifically tackles non-prescription antibiotic use in seven European Union Member States found that: Romania (10%), similar to Greece (12%) and Cyprus (11.7%), had the highest percentages of patients who had used antibiotics without a prescription, reported in the last 18 months of all interviewed respondents, while European Commission (2016) found different percentages: Greece (20%), Romania (16%) and Cyprus (14%) but reported for the last course of antibiotic used (European Commission, 2016; Paget et al., 2017).

Even if the MRG group has better medical knowledge than the SMUG group, they still practice self-medication without prescription, though they know very well the consequences of irrational use.

Requesting an antibiotic to treat a common cold is an inappropriate practice. In our study, 20% of the SMUG group stated that they use this practice. In the general population from Romania, other studies found that antibiotics were considered effective against cold and flu by 39% of the subjects included in the survey in 2016 (European Commission, 2016), compared to 51% of the subjects included in the survey in 2009 (European Commission, 2009) and against bronchitis by 34% of the subjects included in the survey in 2017 (Paget et al., 2017). Previous studies have shown that the percentage of patients asking for antibiotics for common cold treatment, varied depending on the population (14% in Denmark patients ask the physician for antibiotics for upper respiratory infection (Bagger et al., 2015), 10.5% in Germany (Faber et al., 2010) or on the diagnosis (ear ache was the symptom for which 45% of parents expected to receive antibiotics versus common cold with 4.5% of parents (Panagakou et al., 2011).

In the SMUG group, 45% and 26% incorrectly identified bacteria as the cause of common cold and the antibiotics as being effective against viral infections, respectively. Many studies identified that the public generally lacked appropriate knowledge about ABU and ABR and this generated negative attitudes toward the rational use of antibiotics (Ling et al., 2011; Lim & Teh, 2012). In the last years, education through mass media increased the knowledge on ABR: only 7% in the SMUG group and 5% in the MRG group, incorrectly answered that ABR can result after inappropriate use of antibiotics outside the doctor’s indications (Q32).

The results of our study revealed an important level of confusion related to the level of knowledge: in the SMUG group, 93% knew that ABR can result after inappropriate use of antibiotics outside the doctor’s indications, but 62% did not consider that missing an antibiotic dose during treatment as a possible cause of ABR. The impact of the knowledge on the awareness about ABR came up also from the questions with predictive value: Q13, Q14, Q20, Q26 and Q28.

The model generated by multiple logistic regression analysis for the potential prediction of self-medication with antibiotics at any time in life showed that it depended on the ABU practice and ABU knowledge for the SMUG group and on ABR perception for the MRG group.

On the other hand, the potential prediction of self-medication with antibiotics in the month previous to the date of the study was based on questions that belonged, in the SMUG group to: ABU practice, ABR perception and ABR knowledge and in the MRG group to: knowledge about ABR.

Bacterial ABR rates are high in communities with intense practice of non-prescription antibiotics use (Morgan et al., 2011; Zarb & Goossens, 2012) because of very short antibiotic courses, inappropriate drug and dose choices, and unnecessary therapy (Woappi et al., 2016).

The development of ABR is considered one of the most serious problems of the global public health (Boucher et al., 2009; Spellberg et al., 2008), therefore the awareness about the long term consequences of irrational ABU should be increased.

Confusion regarding the perception of ABR as a future problem for medical practice (67%), as a hospital (65%) or as a national problem (60%) was noticed in the SMUG group, but the confusion about ABR as a future problem for medical practice is present among the MRG group as well (16%). On the other hand, ABR was correctly considered only by the MRG group as a problem at national level at 95% and 99% at the hospital level. Other studies found that 78% to 97% of healthcare workers (including medical students, residents, and physicians) perceived ABR as a national problem or as global problem (82%), while 38% to 93% perceived ABR as a hospital problem (Alothman et al., 2016; Giblin et al., 2004; Quet et al., 2015; Remesh et al., 2013; Srinivasan et al., 2004; Wester et al., 2002).

The confusion regarding the influence of ABR on future medical practice could be the result of the lack of knowledge or awareness about a piece of information like, for example, that missing even a single dose of antibiotic can increase the tendency of bacterial resistance to that antibiotic in a person for up to a year (Costelloe et al., 2010).

The SMUG group does not have sufficient appropriate knowledge to understand the measures taken by the physician to prevent ABR, therefore some of them have inappropriate attitudes toward this type of measures. On the other hand, the experience gained during the medical practice and the appropriate medical knowledge, proved that the MRG group had an appropriate attitude in terms of prevention of bacterial ABR, except for the situation that antibiotic cannot be prescribed to a feverish patient if there are no severity criteria.

The antibiotic overuse and the antibiotic self-medication in the community may result from a complex interaction between several factors depending either on the physician or on the patient. The physician’s practice and knowledge, patient’s attitudes, perceptions, beliefs and knowledge on the ABU, patient-physician relationship, patient’s expectations and experience with antibiotics have been studied more frequently (Cockburn & Pit, 1997; Davey, Pagliari & Hayes, 2002; Gould, 2008). Our study shows that subjects who practice self-medication with antibiotics, the SMUG group, had an average basic knowledge regarding the ABU and ABR, but are not aware and maybe do not understand the long-term consequences of the inappropriate use of antibiotics. Their accurate knowledge score for the SMUG group is lower (2.09 ± 1.1) compared with the MRG group (3.58 ± 0.6; *p* < 0.05) and this was one of the factors that influenced self-medication behavior.

Subjects from the general population are not always aware about the importance of compliance in the case of antibiotic treatment of the correct use of antibiotics and are sometimes confused (Skliros et al., 2010; Damian, Lupuşoru & Ghiciuc, 2014; Zoorob et al., 2016; Gonzalez-Gonzalez et al., 2015), therefore educational programs should be instated to inform about the harm produced by administering antibiotics in the most common viral infections. It is essential to increase the awareness of the importance for correct use of antibiotics, not using antibiotics in common viral diseases (i.e., common cold, upper respiratory tract infections etc.), both by healthcare workers and the general population (Gualano et al., 2015; Pulcini et al., 2011; Lv et al., 2014).

There is a need to make the general population more aware, in the sense of increasing the general awareness about the long term consequences of inappropriate use of antibiotics. Even though population’s knowledge about ABU and ABR has improved progressively in the last years, we consider more important that each person should become aware and fully understand the consequences of inappropriate use of antibiotics.

Up to now, most educational programs have been targeted to both physicians and adult public, but more efforts are necessary to change deeply acquired perceptions and attitudes about ABU both in adult population and medical professionals, because they already possess a set of pre-established knowledge and attitudes.

In the last years, as a consequence, apparently, to a variety of programs directed to children education about antibiotic prudent use, the adult population has achieved a good level of basic knowledge regarding antibiotics, but confusion among information regarding the efficacy of antibiotics only against bacteria still needs to be clarified (Hounsa, Kouadio & De Mol, 2010; Buke et al., 2005; Yang et al., 2016).

**The strengths of our study** are that the questionnaires excluded personal identification information and there were no incentives for the participants, which diminished the tendency of the respondents to provide “socially desirable” answers. In addition, the study was conducted during the winter months in which there is usually an increase in the use of antibiotics for infections treatment.

**Limitations of the study** should be kept in mind when interpreting the results. The primary limitation is the design that was used, in the sense that cross-sectional studies do not permit ascertaining causal inferences for the effects of the dependent variables on the outcomes. Second, this study utilized a sample of self-medication user group from one Romanian region and concern about generalizability of the results may arise. This population, compared to the general population, probably underestimates people on self-medication of any type and excludes those on antibiotic medication with prescription. Third, this study utilized a sample of self-identified participants on self-medication with antibiotics, which is vulnerable to selection bias as those in the group with strong interest in this topic might have preferentially accepted to enter the study. Fourth, the participant self-reported the information, with the inability of the researchers to validate with objective measures the answers and some may have overreported socially desirable attitudes and/or behaviors or underreported socially undesirable attitudes and/or behaviors. The absence of identifying data on the questionnaire sheets would tend to minimize such bias. Fifth, as in all survey research, those who did not filled completely the survey may have beliefs and behaviors that differ from those that fully responded.

## Conclusions

The reduced awareness about ABR is revealed by the reduced number of subjects in the SMUG group who don’t know that missing an antibiotic dose during a medical treatment contributes to ABR, comparative with the MRG group. Another question that indicates a low degree of awareness, regards the contribution to ABR of the use of antibiotics no matter for what purpose, outside the indication of a doctor; to this question, both groups have similar frequencies of correct answers (43%).

Indirectly, low ABR awareness in the SMUG group is revealed by the confusion about the appropriate use of antibiotics in bacterial or viral infections (that antibiotics are not used against viruses).

The findings from our study on the perceptions, knowledge and attitudes about ABR in self-medication users might help the policy-makers and regulatory authorities to develop educational programs directed to change the perceptions and attitudes about the appropriate use of antibiotics and diminish self-medication practices with antibiotics. It is a long way to achieving an increase of the awareness about ABR in the general population, especially in self-medication users for antibiotics.

##  Supplemental Information

10.7717/peerj.3803/supp-1 Supplemental Information 1 Questionnaire

10.7717/peerj.3803/supp-2 Supplemental Information 2 Database

## Additional Information and Declarations

### Data Availability

The following information was supplied regarding data availability: The raw data are uploaded as Supplemental Files.
