# Supplementary material for: Awareness about antibiotic resistance in a self-medication user group from Eastern Romania: a pilot study
Source: PeerJ. 2017 Sep 12;5:e3803. doi: 10.7717/peerj.3803 (PMC5600173; doi:10.7717/peerj.3803)
Supplement: Supplemental Information 1 [file peerj-05-3803-s001.pdf]

Dear Sir / Madam, through this questionnaire, we invite you to participate in a study on antibiotics - attitudes and perceptions among patients.
Before completing this questionnaire, you will be given explanations and you can ask questions.
Please read the questions carefully and answer as accurately and completely as possible. This questionnaire is anonymous.
Thank you!

Questionnaire concerning the use of antibiotics - attitudes and perceptions

Fields marked with * are mandatory.

Q1 ***Your age:** _____ years

Q2 ***Sex:**
- ○ Male
- ○ Female

Q3 ***Place of residence:**
- ○ Rural
- ○ Urban

Q4 ***Occupation: ....**
You live in town / village **……………………………........ , county ……………….............**

Do you currently have a chronic condition? **yes** ☐ **no** ☐
***If yes, please indicate*:**

Diabetes mellitus ☐ *Cardiac disease* ☐ *Chronic hepatitis* ☐
Pulmonary Disease ☐ *Dyslipidemia* ☐ *Stroke* ☐
Anxiety / Depression ☐ *Cancer* ☐ *Obesity* ☐

Others:……………………………………………………………………

Q5 * When you fall sick, you appeal to:

- ○ Family doctor
- ○ Another specialist
- ○ Specialist in alternative medicine
- ○ Emergency Room from public hospitals:
- ○ Pharmacist
- ○ Self-medication

Q6 * *How often do you check your health status?*

Monthly	Every 3 months	Yearly	Occasional	When necessary
○	○	○	○	○

Q7 * *How do you appreciate your overall health status?*

Excellent	*Good*	*Medium*	*Poor*	*Bad*
○	○	○	○	○

Dear Sir / Madam, through this questionnaire, we invite you to participate in a study on antibiotics - attitudes and perceptions among patients.
Before completing this questionnaire, you will be given explanations and you can ask questions.
Please read the questions carefully and answer as accurately and completely as possible. This questionnaire is anonymous.
Thank you!

Answer the following questions by **YES** or **NO:**

**Questions:**	**YES**	**NO**
*Q8: Self-medication with antibiotics at any time in life.	○	○
*Q9: Self-medication with antibiotics in the last month.	○	○
*Q10: Compliant with the physician recommendation regarding the antibiotic treatment.	○	○
*Q11: Return to the physician after the antibiotic treatment.	○	○
*Q12: Demand an antibiotic treatment for a common cold.	○	○
*Q13: Demand an antibiotic treatment for a dental infection.	○	○
*Q14: Interested to ask for information about the prescribed antibiotic.	○	○

Answer the following questions by **YES**, **NO** or **I don't know**:

**Questions:**	**YES**	**NO**	**I DON'T KNOW**
*Q15: Antibiotic administration may induce adverse effects.	○	○	○
*Q16: Some antibiotics may induce malformations.	○	○	○

Answer the following questions by **TRUE** or **FALSE**:

**Questions:**	**True**	**False**
*Q17: Bacteria may cause common cold.	○	○
*Q18: Antibiotics are efficient for the treatment of bacterial infections.	○	○
*Q19: Antibiotics are efficient for the treatment of viral infections.	○	○
*Q20: Antibiotics are efficient for the treatment of both bacterial and viral infections.	○	○

Dear Sir / Madam, through this questionnaire, we invite you to participate in a study on antibiotics - attitudes and perceptions among patients.
Before completing this questionnaire, you will be given explanations and you can ask questions.
Please read the questions carefully and answer as accurately and completely as possible. This questionnaire is anonymous.
Thank you!

*Q 21: The usual duration of antibiotic treatment in pneumonia is:

- ☐ less than 3 days
- ☐ 3-7 days
- ☐ more than 7 days

* Q22: Which of the following conditions needs antibiotic treatment:

☐ cough ☐ chills ☐ inflammation ☐ dental infection ☐ throat ☐ fever ☐ neoplasia

Answer the following questions by **YES**, **NO** or **I don't know**:

Questions:	YES	NO	I DON'T KNOW
*Q23: Do you believe that the antibiotics you will receive might contribute to antibiotic resistance?	○	○	○
*Q24: As a future problem for the medical practice.	○	○	○
*Q25: Do you think that it is better to take less antibiotics than those prescribed?	○	○	○
*Q26: As a national problem.	○	○	○
*Q27: As a problem for Romanian hospitals.	○	○	○
*Q28: Missing an antibiotic dose contributes to antibiotic resistance.	○	○	○

Answer the following questions by **TRUE** or **FALSE**:

Questions:	True	False
*Q29: Antibiotic resistance is a result of insufficient knowledge about antibiotic use.	○	○
*Q30: Antibiotic resistance defined as if taken too often, antibiotics will become less and less effective in the future.	○	○
*Q31: Antibiotic resistance is caused by the overuse of antibiotics.	○	○
*Q32: Antibiotic resistance can result after inappropriate use of antibiotics outside the doctor's indications.	○	○

How do you appreciate that the following statements could prevent the development of antibiotic resistance?

**Questions:**	Total agree	Partial agree	Neutral	Partial disagree	Total disagree
*Q33: Wait for the laboratory results in severe infections	○	○	○	○	○
*Q34: Administer an antibiotic in a febrile patient unless severity criteria are present	○	○	○	○	○
*Q35: Accept the most frequent recommended antibiotic	○	○	○	○	○
*Q36: Respect the dosage and timing for the administration of the prescribed antibiotic	○	○	○	○	○

Thank you for collaboration!
